# The Epigenetic Landscape of Borderline Personality Disorder: Insights from a Systematic Review

**DOI:** 10.3390/jcm14228182

**Published:** 2025-11-18

**Authors:** Bartosz Dawidowski, Łukasz Franczak, Piotr Podwalski, Anna Michalczyk, Aleksandra Łupkowska-Grygorcewicz, Oliwia Piotrowska, Jerzy Samochowiec

**Affiliations:** 1 Department of Psychiatry, Pomeranian Medical University, Broniewskiego 26 Street, 71-460 Szczecin, Poland; bartosz.dawidowski@pum.edu.pl (B.D.); lukasz.franczak@pum.edu.pl (Ł.F.);; 2 Department of Hematology and Transplantology, Pomeranian Medical University, 71-252 Szczecin, Poland

**Keywords:** borderline personality disorder, epigenetics, DNA methylation, childhood adversity, stress regulation, neuroplasticity, biomarkers, candidate gene, epigenome-wide association study

## Abstract

**Background/Objectives:** Borderline personality disorder (BPD) is a serious psychiatric condition characterized by affective instability, impulsivity, and self-harming behaviors. Increasing evidence suggests that epigenetic mechanisms, especially DNA methylation, may mediate the interaction between genetic susceptibility and adverse environmental factors. This systematic review aimed to synthesize available findings on DNA methylation in BPD, including candidate gene studies and epigenome-wide association studies (EWAS). **Methods:** We conducted a systematic search of PubMed, Embase, and Scopus databases following PRISMA guidelines. Eligible studies (*N* = 19) included original research examining DNA methylation in individuals with BPD, assessed either through candidate gene approaches or genome-wide platforms. Data were extracted regarding study design, sample characteristics, psychometric instruments, genes, CpG sites analyzed, and main findings. **Results:** Inconsistent associations were found between BPD and altered methylation of several candidate genes, such as NR3C1, FKBP5, BDNF, DRD2, HTR2A, and COMT. Differential methylation was often linked to early-life adversities and symptom severity. EWAS also identified new loci, including APBA3, MCF2, PXDN, and OPRK1. Across studies, methodological heterogeneity and small sample sizes limited definitive conclusions. **Conclusions:** Evidence for DNA methylation alterations in BPD is mixed, and current findings do not allow firm conclusions about their mechanisms or clinical relevance. Larger and longitudinal studies are required to clarify whether these epigenetic changes contribute meaningfully to BPD.

## 1. Introduction

Borderline personality disorder (BPD) is a severe psychiatric condition characterized by pervasive emotion dysregulation, identity disturbance, and unstable interpersonal functioning [1]. It affects about 0.7–2.7% of adults in the general population, with markedly higher prevalence in clinical settings [2]. BPD is linked to significant functional impairment, high rates of self-harm and suicidal behavior, and substantial societal costs [2]. Although it has traditionally been seen as highly challenging to treat, recent research and clinical advances have led to earlier diagnosis, more effective psychotherapeutic interventions, and better long-term outcomes [1].

Epigenetic mechanisms serve as a vital link between environmental exposures and gene expression, thus playing a key role in the development of psychiatric disorders. DNA methylation, the most extensively studied epigenetic modification, can be dynamically affected by stress, trauma, and developmental factors, influencing neurobiological vulnerability throughout the lifespan [3,4]. Meta-analyses have shown that altered methylation patterns are common across various neuropsychiatric disorders, including depression, schizophrenia, and bipolar disorder, frequently involving stress-related genes and pathways [5,6]. Such modifications may last over time, influencing the long-term effects of early-life adversity on psychopathology. Still, they also remain potentially reversible, emphasizing their importance as biomarkers and therapeutic targets in mental disorders [3,4].

Porter et al. meta-analytically demonstrated that patients diagnosed with BPD have nearly a 14-fold higher risk of experiencing childhood adversity than healthy controls. Although prospective and epidemiological studies have shown a lower risk—2.6 times higher than in control groups—the link between early life stress and the development of BPD is well documented in the literature, and over 70% of patients report some form of adversity in childhood [7]. Certain types of childhood adversities, especially those involving abuse or sexual characteristics, can affect the functioning of the hypothalamic–pituitary–adrenal axis (HPA) by causing long-term increases in the cortisol awakening response and basal cortisol levels, as shown in both hair and blood measurements and observed in both clinical and non-psychiatric populations [8,9]. Similarly, in BPD, there are disruptions in the functioning of the HPA axis, evidenced by elevated continuous cortisol levels and a blunted response to psychosocial stressors [10]. Changes in NR3C1 gene methylation, which encodes the glucocorticoid receptor protein, have been linked to variations in its expression [11]. These changes may affect the regulation of the HPA axis as well as the psychopathology of mental disorders [11], making this gene a natural focus for research within the BPD population.

Another important focus in this research area is brain-derived neurotrophic factor (BDNF). BDNF is a member of the neurotrophin family that can be secreted not only by neurons but also by other cell types, including leukocytes, endothelial cells, and platelets [12]. BDNF plays a crucial role in neuroplasticity, neuronal migration, cell survival, and maintaining synapses [13]. Reduced peripheral BDNF levels have been consistently reported in patients diagnosed with major depressive disorder, bipolar disorder, schizophrenia, obsessive compulsive disorder, generalized anxiety disorder, and panic disorder [13]. Conversely, significantly elevated BDNF levels have been observed in individuals with post-traumatic stress disorder [13]. In major depressive disorder and bipolar disorder, there is additional evidence supporting BDNF as a potential biomarker of treatment response [12]. Moreover, BDNF levels not only increase in response to psychosocial stress but also show an association with HPA axis activity: higher cortisol reactivity to stress is linked to a slower return of BDNF concentrations to baseline [14].

For reasons outlined above, current epigenetic research in BPD mainly focuses on genes involved in stress response and neuroplasticity, especially NR3C1 and BDNF. Changes in methylation of NR3C1 have been consistently linked to HPA axis dysregulation and childhood adversities, with most studies noting hypermethylation in patients who experienced severe abuse [15,16,17]. Similarly, increased BDNF methylation has been linked to clinical severity and early life stress [18,19]. Besides these two genes, methylation differences have also been found in others, such as those involved in monoaminergic (DRD2, COMT, HTR2A, MAOA/B) and opioid (OPRK1) signaling, as well as in new candidates identified by epigenome-wide studies, including APBA3, MCF2, PRIMA1, and PXDN [20,21,22,23,24].

Despite converging evidence, the field remains characterized by methodological heterogeneity and inconsistent findings. Studies differ in scope and the specific CpG sites targeted, resulting in reports of hyper- or hypomethylation for the same genes across cohorts that have often been used in more than one study [21,22,23,25,26,27]. Furthermore, many of the implicated genes exhibit similar changes in other psychiatric disorders, raising questions about their specificity to BPD [4]. Together, these observations emphasize both the potential and current limitations of epigenetic research in BPD, highlighting the need for systematic synthesis to find methylation patterns specific to BPD.

We aimed to systematically synthesize evidence on DNA methylation changes in BPD. Specifically, we sought to: (1) compile genes and CpG regions studied to date; (2) summarize the direction and extent of methylation differences between BPD and healthy or other psychiatric comparison groups; (3) evaluate links between methylation, childhood adversities, and clinical phenotypes (such as symptom severity, suicidality, and psychotherapy response); and (4) pinpoint common, potentially disorder-specific markers and significant research gaps.

## 2. Materials and Methods

We conducted a systematic review of studies examining DNA methylation alterations in borderline personality disorder (BPD) following prior registration in PROSPERO (CRD420251108003). The authors conducted a systematic search across three databases, adhering to the PRISMA 2020 guidelines for reporting systematic reviews.

### 2.1. Literature Search

The search was conducted using the PubMed, Embase, and Scopus databases. Search terms were primarily limited to English-language keywords, and the search focused on studies involving human participants. Medical Subject Headings (MeSH) were utilized. Keywords included: borderline personality disorder, emotionally unstable disorder, childhood trauma, childhood maltreatment, adverse childhood experiences, adult survivors of child abuse, epigenetics, epigenome, epigenomics, DNA methylation, targeted bisulfite sequencing, and neuropsychiatric genes. Abbreviations were also considered.

### 2.2. Inclusion and Exclusion Criteria

Original articles published before 18 July 2025 were included if they met the following criteria: (1) A sample of patients aged over 18 and under 70 with a diagnosis of borderline personality disorder (according to DSM-IV, DSM-5, ICD-10, or ICD-11); (2) Assessment of DNA methylation levels at the gene or genome level using quantitative methods; (3) Comparison of DNA methylation levels in selected genes between borderline personality disorder and healthy controls or another psychiatric group, or reporting of correlations with clinical variables and adverse events/childhood trauma within the BPD population; (4) Observational (longitudinal) or cross-sectional study design; (5) Publication written in English.

Exclusion criteria were as follows: (1) Non-original data (case studies, conference abstracts, systematic or non-systematic reviews, etc.); (2) No participants with a BPD diagnosis included; (3) Non-quantitative methods used for methylation assessment; (4) Languages other than English.

### 2.3. Data Extraction

Two independent researchers, B.D. and Ł.F., initially selected records for inclusion. In cases of disagreement, another author, P.P., made the final decision. We verified the eligibility of search results twice, first by title and abstract, and then by full text. The reasons for excluding each study were documented. A manual search of references in downloaded papers was conducted, and we also checked the “Cited by” sections of record websites when available. The primary researcher used a Microsoft Excel spreadsheet to gather important data from each study.

The data extracted from the included studies included: authors, year of publication, methodology, and socio-economic and medical indicators used in the original analyses (sample size and characteristics such as age range, gender distribution, substance use, medication status), as well as any coefficients/descriptive statistics needed to analyze the relationship between methylation, BPD diagnosis, clinical variables, and childhood adversities.

On 26 July 2025, a preliminary search identified 125 records after removing duplicates, entries not in English, books, theses, dissertations, and non-peer-reviewed publications. We conducted an initial search on 30 July 2025, which yielded an additional 8 records. Two members of the review team independently reviewed each record during the abstract screening. If the authors disagreed on which study to include, the other author made the final decision. Articles were included only if they met the specified inclusion and exclusion criteria. As a result, 19 articles were deemed appropriate for inclusion in the review.

### 2.4. Quality Assessment

The methodological quality of the included studies was assessed using an adapted version of the *Newcastle–Ottawa Scale* (NOS) for case–control studies. Given that most studies investigated DNA methylation differences between individuals with BPD and healthy controls, the “exposure” domain was reinterpreted as the accuracy of BPD diagnosis. Each study was rated on three domains: selection (0–4), comparability (0–2), and exposure/outcome assessment (0–3). Studies scoring ≥7 were considered high quality, 4–6 moderate quality, and ≤3 low quality. Two independent reviewers assessed all studies; disagreements were resolved by discussion. The complete scoring table is provided in Table 1.

## 3. Results

### 3.1. Study Selection

**Figure 1:**
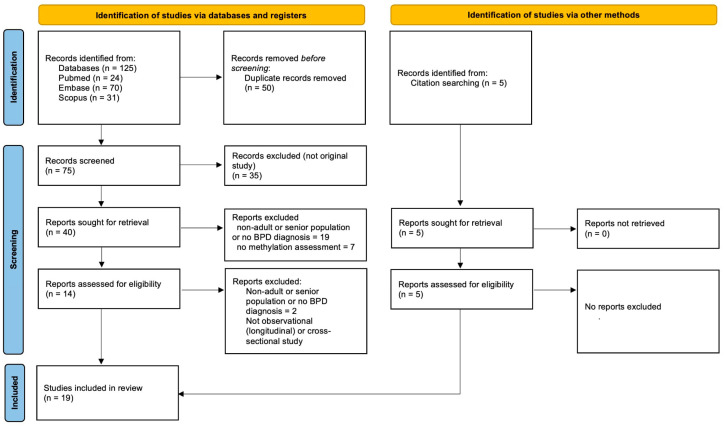
PRISMA flowchart for the inclusion of studies.

We identified 125 studies through PubMed, Embase, and Scopus, of which 50 were duplicates, and an additional 5 records were retrieved through citation searching (Figure 1). Sixty-one studies were excluded at the title and abstract screening stage, and 3 more were excluded after full-text assessment. Ultimately, 16 studies published between 2011 and 2024 were included in this review. Fourteen of the eligible studies investigated participants with a diagnosis of BPD, and 2 assessed participants with bulimia nervosa stratified by the absence or presence of comorbid BPD (Table 2). The studies examined a wide range of genes, with the most prominent being NR3C1, FKBP5, and BDNF. Three of the included studies were epigenome-wide association studies (EWAS). Sample sizes ranged from 35 to 547 total participants and from 14 to 389 patients diagnosed with BPD. Only 5 studies included male participants, with most of these representing very small subgroups derived from the same cohorts as in other studies.

### 3.2. Quality Assessment

Across the 19 included studies, the overall methodological quality ranged from low to moderate, with no study achieving a high-quality rating (≥7/9). The most common total scores were 4–6 points, indicating moderate quality in most publications. In contrast, several earlier studies scored only 3/9 points, reflecting *low* quality due to incomplete reporting and limited comparability between groups.

Most studies received two or three stars in the Selection domain, indicating clear diagnostic definitions and fairly representative case recruitment. However, the lack of detailed information on recruitment processes or response rates limited higher scores in this area. The Comparability domain showed varied results: about half of the studies controlled for key confounders such as age, sex, or comorbidities, while others offered insufficient details on matching or statistical adjustments. The Exposure domain was generally consistent, with most studies meeting the basic standard of comparable data collection procedures between cases and controls.

Among the better-rated studies (e.g., Steiger et al., Groleau et al., Arranz et al., Perroud et al.), comparability and completeness of participant data were higher, supporting moderate overall quality [18,20,23,28]. By contrast, earlier reports (e.g., Dammann et al., Teschler et al., Moser et al.) lacked clarity regarding participant retention or control matching, thus receiving low scores [21,26,27].

In summary, the assessment of the methodology shows that the current literature has moderate overall quality, mainly limited by incomplete reporting of participant flow and control matching rather than by diagnostic or design flaws. Future research should focus on more transparent reporting of sample recruitment and participant retention to reduce selection bias and enhance comparability across studies.

### 3.3. Intervention Characteristics

The three EWAS included in this review used Infinium BeadChips to examine genome-wide DNA methylation [23,25,27]. Two of these, along with the other studies reviewed, utilized standard bisulfite pyrosequencing [15,16,17,20,21,22,23,24,26,27,28,29,30,31,32,33,34]. However, one study additionally measured methylation using the COBRA (Combined Bisulfite Restriction Analysis) method before pyrosequencing, and another employed a high-resolution melt assay [18,21]. Across all the studies, DNA sources included peripheral leukocytes, whole blood, or saliva. The investigations evaluated both the average methylation of specific genomic regions and methylation levels at individual CpG sites.

Bisulfite pyrosequencing is regarded as the gold standard for DNA methylation analysis, enabling efficient and consistent measurement of methylation at individual CpG sites (5′—cytosine—phosphate—guanine—3′) within targeted DNA regions. The process includes several steps: (1) bisulfite conversion, which maintains methylated CpGs while converting unmethylated cytosines, (2) designing PCR primers specific to the bisulfite-converted DNA region of interest, (3) PCR amplification, (4) purifying and isolating single DNA strands, (5) hybridizing primers during the pyrosequencing reaction, and (6) analysing sequencing results with specialized software. Compared to COBRA, which depends on restriction enzymes, bisulfite pyrosequencing provides greater accuracy and can assess a larger number of CpG sites [35].

Infinium BeadChips enable the analysis of DNA methylation at individual CpG sites on a genome-wide scale and are based on the following steps: (1) hybridization of bisulphite-converted and amplified DNA fragments to 50-mer oligonucleotides attached to each bead; (2) single-base extension at the 3′ end of the oligonucleotide with labelled ddNTPs, followed by fluorescent staining of the incorporated ddNTPs; and (3) measurement of the fluorescence intensity of each bead. Although this method targets only about 3% of all CpG sites in the genome, it offers an efficient way to evaluate DNA methylation levels in large populations [36].

**Table 1:** Quality assessment of the included studies.

Study	Selection	Comparability	Exposition	Sum	Quality
Gescher et al. [30]	★	★★	★	4/9	Moderate
Edelmann et al. [24]	★	★★	★	4/9	Moderate
Prados et al. [25]	★★	-	★	3/9	Low
Perroud et al. [18]	★★★	★★	★	6/9	Moderate
Flasbeck et al. [17]	★★	★	★	4/9	Moderate
Martín-Blanco et al. [16]	★	★★	-	3/9	Low
Perroud et al. [34]	★★	★★	★	5/9	Moderate
Steiger et al. [28]	★★★	★★	★	6/9	Moderate
Groleau et al. [20]	★★★	★★	★	6/9	Moderate
Arranz et al. [23]	★★★	★★	★	6/9	Moderate
Perroud et al. [15]	★★	-	★	3/9	Low
Jamshidi et al. [19]	★★	★★	★	5/9	Moderate
Moser et al. [26]	★★	-	★	3/9	Low
Damman et al. [21]	★	★	★	3/9	Low
Teschler et al. [27]	★	★	★	3/9	Low
Teschler et al. [22]	★	★	★	3/9	Low
Thomas et al. [33]	★★	★★	★	5/9	Moderate
Thomas et al. [29]	★★	★★	★	5/9	Moderate
Knoblich et al. [31]	★★	★★	★	5/9	Moderate

★ = one point awarded (meets NOS criterion); ★★ = two points awarded (meets two NOS sub-criteria); ★★★ = three points awarded (meets all sub-criteria within the domain). Maximum scores: Selection (0–4), Comparability (0–2), Exposure (0–3).

**Table 2:** Table with selected characteristics of included (N = 19). BPD—borderline personality disorder, HC—health controls, MDD—major depressive disorder, SAD—social anxiety disorder, ADHD—attention deficit hyperactive disorder, PTSD—posttraumatic stress disorder, AUD—alcohol use disorder, SUD—substance use disorder, BD—bipolar disorder, SchAD—schizoaffective disorder, AnxD—anxiety disorders, DE—depressive episode, ME—mood episode, PME—past psychotic mood disorder, CTQ—Childhood Trauma Questionnaire, CTI—Childhood Trauma Adversities, FT—Fagerstrom Test.

Study	Year	Sample					DNA Source	Trauma Measure	Clinical Measures	Genes (CpGs)	Main Findings
		N	Females	Age	BPD Comorbidities	Medication					
Gescher et al. [30]	2024	BPD = 47, HC = 48	BPD = 47, HC = 48	BPD 25.21 ± 4.21, HC 24.71 ± 3.96	Not reported	BPD = 0, HC = 0	whole blood samples	CTQ	BSL-23, BIS, DES -FDS, ZAN-BPD	OPRK1	Identified a novel OPRK1 promoter region (five CpGs, a DMR) with lower mean methylation in BPD vs. controls. Lower methylation of this OPRK1 region also correlated with higher BPD symptom severity and with greater trait impulsivity and was significantly associated with higher childhood emotional neglect.
Edelmann et al. [24]	2024	BPD = 40, HC = 53, MDD = 64, HC = 64, SAD = 65, HC = 72	BPD = 33, MDD = 26, SAD = 45, HC (BPD) = 46, HC (MDD) = 17, HC (SAD) = 34	BPD: 31.6, MDD: 38.6, SAD: 25.9, HC(BPD): 28.3, HC(MDD): 25.9, HC(SAD): 24.71	Not reported	Not reported	whole blood samples	CTQ	ELA	PXDN exon cg10888111 (chr:2 1,632,996–1,633,597)	In BPD patients with high early-life adversity (ELA), methylation at a PXDN gene CpG (cg10888111) was significantly lower than in BPD with low ELA or controls.
Prados et al. [25]	2015	BPD = 95, MDD = 93	BPD = 88, MDD = 59	BPD: 32.17 ± 0.99, MDD: 41.39 ± 1.37	AUD = 52, SUD = 43	BPD = 73, MDD = 70	whole blood samples	CTQ	None	genome-wide methylation analysis	The top hit (cg04927004, near miR-124-3) was significantly hypomethylated in BPD (0.22 in BPD vs. 0.35 in controls). Other significant sites (e.g., near WDR60, FAM163A) also had lower methylation in BPD. Many top DMRs were on the X chromosome (more methylated in BPD) and on chromosome 6.
Perroud et al. [18]	2013	BPD = 115, HC = 52	BPD = 108, HC = 24	BPD: 30.36 ± 9.19, HC: 40.65 ± 12.04	BD = 22, MDD = 84, SchAD = 9	BPD = 55 (Antidepressants)	peripheral blood leukocytes	CTQ	BDI, BHS, BIS-10, SCID-II BPD part	BDNF CpG exons I and IV	BPD patients had higher methylation of BDNF promoters (exons I and IV), and individuals with more severe childhood trauma had higher methylation. After 12 weeks of DBT, mean BDNF methylation increased overall, driven by non-responders, while therapy responders showed a decrease.
Flasbeck et al. [17]	2021	BPD = 45, HC = 44	BPD = 45, HC = 44	BPD: 26.3 ± 5.7, HC: 24.0 ± 3.1	DE = 23, PTSD = 8, AnxD = 2, SUD = 13	Antidepressant = 18, Antipsychotic = 2, Antidepressant and Antipsychotic = 8, Anticonvulsants = 2, Other psychoactive drugs = 1	mouthwash samples, saliva	CTQ	IRI, SCI-90-R	1F promoter of NR3C1 and the intron 7 of FKBP5	Significantly lower mean exon 1F methylation in BPD compared to HC. Still, despite statistical significance, the difference between groups was smaller than the sensitivity of the methylation analysis method used
Martín-Blanco et al. [16]	2014	BPD = 281, HC = 0	BPD = 239	BPD: 29.4 ± 7	SUD = 155, AnxD = 131, AUD = 100, ED = 81	BPD = 247	peripheral blood leukocytes	CTQ-SF	None	exon 1F of NR3C1	NR3C1 promoter methylation (exon 1F) correlated positively with childhood abuse and with illness severity. Specifically, patients with more severe physical abuse and higher severity scores and hospitalizations showed higher NR3C1 methylation.
Perroud et al. [34]	2016	BPD = 116, ADHD = 111, BD = 122	BPD = 106, ADHD = 33, BD = 65	BPD: 31.5 ± 9.74, ADHD: 37.65 ± 10.36, BD: 45.25 ± 11.7	PME = 33, SAD = 67 AUD = 65, MDD = 75, BD = 25	Not reported	peripheral blood leukocytes	CTQ	None	5HT3AR (CpG1 I, CpG2 II, CpG3 II, CpG1 III, CpG2 III, CpG3 III, CpG4 III, CpG5 III)	Altered HTR3A methylation in BPD, with lower levels at CpG1_I and CpG5_III and higher levels at several other sites. In BPD, methylation at specific CpGs correlated with childhood trauma, particularly physical abuse, and CpG2_III and CpG5_III methylation mediated the link between abuse severity and suicidality, hospitalizations, and mood episodes.
Steiger et al. [28]	2013	BN + BPD = 14, BN-nBPD = 47, HC = 32	BN = 64, (BN + BPD = 14, BN-nBPD = 47), NED = 32	BN: 26.05 ± 6.59, NED: 23.67 ± 5.70	Not reported	BN = 33 NED = 0	whole blood samples	CTI	SCID-I, DIS4, CAPS, SCID-II BPD part	NR3C1 promoters 1B, 1C, 1F, and 1H	Significantly lower mean exon 1H methylation in the BN + BPD group compared to the BN only and HC groups, and one CpG in exon 1C (chr5:142763355–142763361) showed significantly higher methylation in the BN + BPD group compared to HC
Groleau et al. [20]	2014	BN = 52 (BN + BPD = 8, HC = 19)	BN = 52 (BN + BPD = 8), HC = 19	BN: 24.67 ± 5.68, HC: 23.68 ± 4.57	Not reported	BN = 35, HC = 0	whole blood samples	CTI	SCID II BPD	DRD2	Significantly higher mean methylation across the first 10 CpGs of DRD exon 1 (chr11:113346140–113346389) in BN + BPD compared to HC, with a trend toward higher methylation compared to BN alone
Arranz et al. [23]	2021	BPD = 96, HC = 44, replication cohort BPD = 293, HC = 114	BPD = 96, HC = 44, replication cohort BPD = 293, HC = 114	Replication sample BPD: 30.93 ± 7.2, HC—Not reported	No current episode of any Axis I disorder no severe physical conditions, neurological disease, or mental deficiency	BPD = Not reported, HC = 0	whole blood samples	CTQ-SF	SCID II, DIB-R, MSI-BPD,	genome-wide methylation analysis	Several X-chromosomal CpGs (e.g., in PQBP1, ZNF41, RPL10) and one on chr6 (TAP2) lower methylation in BPD. Hypomethylation differences were amplified in BPD subjects with childhood trauma. Additionally, four autosomal genes (e.g., POU5F1, GGT6) were differentially methylated depending on trauma history.
Perroud et al. [15]	2011	BPD = 101, MDD = 99, MDD + PTSD = 15	BPD = 95, MDD = 64, MDD + PTSD = 11	BPD: 30.76 ± 9.74, MDD: 41.63 ± 12.81, MDD + PTSD: 37.33 ± 10.46	MDD = 74, BP I = 4, BP II = 15, SChAD = 8, AUD = 57, SUD = 48, PTSD = 25	Antidepressant: BPD = 3 9, MDD = 70, MDD + PTSD = 6, Neuroleptics: BPD = 40, MDD = 4, MDD + PTSD = 1, Mood stabilizers: BPD = 14, MDD = 1, MDD + PTSD = 0, None: BPD = 17, MDD = 28, MDD + PTSD = 9	whole blood samples	CTQ	BDI-SF, SCID II, French version of DIGS	exon 1F NR3C1 promoter (CpGs 6–13)	Among BPD subjects, those with childhood maltreatment (especially sexual abuse) had increased methylation of the NR3C1 promoter, with methylation levels positively correlating with abuse severity. Higher NR3C1 methylation also tended to associate with greater clinical severity.
Jamshidi et al. [19]	2023	Discovery cohort: BPD = 97, HC = 32, Validation cohort: 60	Discovery cohort: BPD = 97, HC = 32, Validation cohort: 60	BPD: 29.4 ± 7.6, HC: 37.2 ± 6.0, SA: HR 35.67 ± 13.2, LR 34.0 ± 12.4	Active MDD = 41, Severe MDD = 13, BD = 8, AnxD = 59, AUD = 32, SUD = 26	HC = 0, BPD: SSRI = 32, non-SSRI antidepressants = 20, Mood stabilizers = 4, Benzodiazepines = 35, Neuroleptics = 12	peripheral blood leukocytes	None	SCID I, SCID II	BDNF 16 individual CpG-sites	In women with BPD and severe suicide attempts, mean methylation of a targeted BDNF locus was higher than in controls This elevation was replicated in an independent cohort of female suicide attempters with Borderline/EUPD.
Moser et al. [26]	2020	BPD = 45, HC = 45	BPD = 45, HC = 45	BPD: 26.3 ± 5.7, HC 24.5 ± 4.4	Not reported	Not reported	saliva samples	CTQ	None	42 CpGs from the NR3C1 1 F promoter, 84 CpGs of the SLC6A4, 5 CpGs in FKBP5 intron 7, 12 CpGs intron 3 of the OXTR	No biologically relevant differences in mean methylation were found between groups.
Dammann et al. [21]	2011	BPD = 26, HC = 11	BPD = 24, HC = 11	BPD: 33 ± 11, HC 32 ± 7	AUD = 6, SUD = 4, Mental and Compulsions = 1, MDD = 3, AnxD = 1, Anorexia = 1, Narcissistic personality disorder = 1, ADHD = 1	Not reported	whole blood samples	None	None	14 neuropsychiatric genes (COMT, DAT1, GABRA1, GNB3, GRIN2B, HTR1B, HTR2A, 5-HTT, MAOA, MAOB, NOS1, NR3C1, TPH1 and TH)	A broad hypermethylation pattern in BPD: on average higher methylation across neuropsychiatric genes. In particular, BPD patients had increased promoter methylation at HTR2A, NR3C1, MAOA and the S-COMT locus.
Teschler et al. [27]	2013	BPD = 24, HC = 11	BPD = 24, HC = 11	BPD: 33 + 11, HC: 32 ± 7	As in Damman (2011)	Not reported	whole blood samples	None	None	genome-wide methylation analysis	Significantly higher methylation of APBA3 (cg20366831), KCNQ1 (cg17820828), MCF2 (cg21557231), and NINJ2 (cg20781967) in BPD compared to HC, and confirmed these findings with pyrosequencing
Teschler et al. [22]	2016	BPD = 24, HC = 11	BPD = 24 HC = 11	BPD: 33 + 11, HC: 32 ± 7	As in Damman (2011)	BPD = 24, HC not reported	whole blood samples	None	None	rDNA promoter region, promoter of PRIMA)	In female BPD patients, the PRIMA1 promoter showed hypermethylation and the ribosomal RNA (rDNA) gene showed hypomethylation.
Thomas et al. [33]	2018	BPD = 41, HC = 41	BPD = 3, HC = 35	BPD: 30.4 ± 8.6, HC: 30.7 ± 9.3	Habitual smokers = 22, AUD = 35	BPD = 35, HC = 0	saliva, whole blood samples	CTQ	GSI score (SCL90R), PST score (SCL90R), BSL-23	BDNF IV promoter	BPD patients showed higher methylation at four CpGs in the BDNF exon-IV promoter in saliva (but not in blood) relative to controls. Moreover, after a 12-week psychotherapeutic intervention, salivary BDNF methylation decreased significantly in the patient group.
Thomas et al. [29]	2019	BPD = 44, HC = 44	BPD = 37, HC = 37	BPD: 29.5 ± 8.4, HC: 29.7 ± 8.8	Not reported	BPD = 40, HC = 0	whole blood samples	CTQ	GSI score (SCL90R), PST score (SCL90R), BSL-23, AUDIT, FT	COMT	BPD patients had significantly lower methylation at one CpG (cg19962541) than controls, independent of genotype.
Knoblich et al. [31]	2018	BPD = 44, HC = 44	BPD = 37, HC = 37	BPD: 29.5 ± 8.4, HC: 29.7 ± 8.8	Not reported	Not reported	whole blood samples	CTQ	SCL90R, BSL223	two CpG sites within APBA3 and one CpG site within MCF2	No case–control differences in APBA3 or MCF2 methylation were observed. However, among BPD patients undergoing DBT, those who responded to therapy had significantly higher baseline methylation of APBA3 and MCF2 than non-responders, suggesting these marks predicted treatment outcome.

### 3.4. Psychometric and Childhood Adversity Measures

Several studies in this review investigated the impact of trauma on DNA methylation. Three different tools were used: the Childhood Trauma Questionnaire (CTQ), its short form (CTQ-SF), and the Childhood Trauma Interview (CTI). The CTQ was used in 11 studies, the CTQ-SF in 2, and the CTI in 2 as well. Four of the studies included in this review did not use any tool to assess early-life trauma or childhood adversities.

CTQ is one of the most widely used retrospective self-report instruments for assessing child maltreatment, covering five domains: emotional, physical, and sexual abuse, as well as emotional and physical neglect [37]. In brief, the CTQ-SF was developed and validated as an efficient screening tool, maintaining strong psychometric properties. It features 25 scorable items with high reliability and validity across diverse samples [38]. Both versions utilize a Likert-type scale to assess the severity of the adverse experiences dimensionally, enabling quantitative analysis of maltreatment load [37,38]. In contrast, the CTI is a semi-structured retrospective interview that evaluates six domains: separation or loss of a caregiver, neglect, emotional abuse, physical abuse, witnessing violence, and sexual abuse or assault [39]. The CTI involves interviewer assessments of severity, frequency, and duration, supported by standardized coding manuals and training, thereby improving validity relative to self-report questionnaires [39].

Many included studies also employed various instruments to assess participants’ clinical status, which are summarized in Supplementary Table S1.

### 3.5. NR3C1 and FKBP5

Five studies compared the methylation levels of NR3C1 between BPD and HC or examined correlations between its methylation and childhood adversities or clinical variables in BPD [15,16,17,21,28]. Flasbeck et al. used mouthwash samples as a DNA source, Martín-Blanco et al. used peripheral leukocytes, Steiger et al., Perroud et al., and Dammann et al. used whole blood genomic DNA [15,16,17,21,28]. Three publications considered the same 8 CpGs located within the exon 1F promoter in their analysis [15,16,21]. Flasbeck et al. examined methylation at 42 CpGs also situated within the entire exon 1F, while Steiger et al. chose to perform a more extensive examination of multiple CpGs across all four exons of NR3C1 [17,28].

The study populations were notably diverse in terms of sample size, gender, and age. Flasback et al. and Damman et al. compared relatively small BPD samples to HC (*n* = 89, *n* = 37, respectively) [17,21]. Martín-Blanco et al. studied the largest BPD group (*n* = 281) but did not compare it to another group; instead, they focused on childhood adversities and clinical features [16]. Steiger et al. compared small groups of patients with bulimia nervosa (BN) without BPD (*n* = 47), with bulimia nervosa and BPD (BN + BPD, *n* = 14), and HC (*n* = 32) [28]. Perroud et al. analyzed a larger BPD sample (*n* = 101) and compared it to groups of patients with MDD (*n* = 99) and those with both MDD and PTSD (*n* = 15). Perroud et al. chose to compare the BPD population with MDD due to the significant comorbidity of both disorders. At the same time, the MDD group included only participants with low reported levels of childhood adversity with BPD features. This allowed for comparison of groups with similar levels of depressive symptoms yet differing in the severity of BPD symptoms [15].

Additionally, most studies included either exclusively female or predominantly female participants. Dammann et al. included only two males with BPD, while no males were in the HC group [21]. Similarly, Perroud et al. included six males in the BPD group, none in the MDD group, and 4 in the MDD + PTSD group [15]. Flasbeck et al. and Steiger et al. included all-female groups [17,28]. Only Martín-Blanco et al. included a sizable sample of males (*n* = 42) [16]. The average age of the study populations ranged from 25.2 to 32.7 years. Flasbeck et al. noted a trend toward older age in the BPD group compared to the HC group, whereas Perroud et al. reported a significantly lower age in the BPD group compared to the MDD and MDD + PTSD groups [15,17]. Participants in the BPD groups had significant psychiatric comorbidity with current or past depressive episodes, PTSD, anxiety disorders, and substance use disorders (mainly alcohol and benzodiazepines) being the most common examples. Additionally, most BPD participants were taking some form of psychopharmacotherapy, with antidepressants being the most common.

Flasbeck et al. found significantly lower mean exon 1F methylation in BPD compared to HC. Still, despite statistical significance, the difference between groups was smaller than the sensitivity of the methylation analysis method used [17]. Additionally, they found no significant differences in methylation levels at individual CpGs. Steiger et al. observed significantly lower mean exon 1H methylation in the BN + BPD group compared to the BN only and HC groups, and one CpG in exon 1C (chr5:142763355–142763361) showed significantly higher methylation in the BN + BPD group compared to HC [28]. Perroud et al. detected nominally higher mean exon 1F promoter methylation in BPD compared to MDD and MDD + PTSD. Although the differences were larger than those reported by Flasbeck et al., statistical analysis of between-group differences was not conducted [15]. Damman et al. reported significantly higher mean methylation in BPD relative to HC within the exon 1F promoter, as well as in individual CpGs (designated as CpG1, CpG5, CpG8 by the authors) [21].

Perroud et al. found significant but slightly higher average methylation within the exon 1F promoter in BPD participants who experienced sexual abuse and physical neglect compared to those who did not. Furthermore, the level of average methylation correlated with the number of childhood abuse and neglect experiences [15]. Although Martín-Blanco et al. also observed significant correlations of average methylation within the exon 1F promoter, these were for different types of childhood adversities. In this study, methylation status correlated with the severity of physical abuse, self-harm, stress, and previous psychiatric hospitalizations. A trend toward correlation with emotional neglect was also seen. In the same study, methylation status within three specific CpGs (CpG1, CpG2, CpG3) significantly correlated with physical abuse and emotional neglect. Additionally, the authors found a significant link between methylation at CpG6 and emotional abuse [16]. Flasbeck et al. found nominal correlations between the average methylation within exon 1F and the severity of childhood adversities and their main subtypes. Still, none of the correlations remained significant after correction for multiple comparisons [17].

FKBP5 encodes a cochaperone protein for heat shock protein 90 (Hsp 90) [40,41,42]. Hsp 90 is one of the proteins involved in regulating the translocation of the GR, encoded by NR3C1, from the cytoplasm to the nucleus [40,41,42]. FKBP5 expression is affected by environmental stressors and may therefore downregulate GR activity by influencing Hsp90 [43]. Of the included studies, only Flasbeck et al. examined the methylation status of FKBP5 within intron 7 (chr6: 35558322–35558593). They found no statistically significant differences in the average methylation levels between BPD and HC, nor were there any differences between smaller regions (bin1, bin2). The authors suggested that this may have been due to the study’s low statistical power. Nevertheless, a significant positive correlation was found between methylation and the severity of anxiety symptoms (bin 2 only) and the Global Severity Index (bin 2 and mean methylation) [17].

### 3.6. BDNF

Three of the studies included in this review investigated BDNF methylation changes in BPD compared with healthy controls (HC). Perroud et al. and Jamshidi et al. both used leukocytes as the source of DNA [18,19]. Thomas et al. used both whole blood and saliva samples [33]. Perroud et al. examined 9 CpG sites within exon 1 (chr11: 27743473–27744564) and 17 CpG sites within exon 4 (chr11: 27723060–27723294) [18]. Thomas et al. have analysed a similar region of exon 4 but inspected only 4 CpG (chr11: 27723161, chr11: 27723159, chr11: 27723143, chr11: 27723137) [33]. In contrast, Jamshidi et al. analysed a region located 2000 base pairs upstream of the transcriptional start site, comprising 16 CpG sites [19].

Perroud et al. recruited 115 participants with BPD (*n* = 115), of whom 108 were female (*n* = 108, 93.91%), and 52 HC (*n* = 52), including 24 women (*n* = 24, 46.15%). The HC group was significantly older than the BPD group (mean ages: 40.65 vs. 30.36 years). The BPD cohort presented with substantial DSM-IV Axis I comorbidity, including bipolar disorder, major depressive disorder, and schizoaffective disorder. All BPD participants were receiving psychotropic medication, although the authors did not specify drug classes [18]. By contrast, Jamshidi et al. studied exclusively female samples of BPD (*n* = 97) and HC (*n* = 32). As in the Perroud study, the HC group was significantly older than the BPD group (mean ages: 37.2 vs. 29.4 years). The BPD group also showed high rates of comorbidity, including major depressive disorder, bipolar disorder, anxiety disorders, and histories of alcohol or substance misuse. A substantial proportion of the BPD group was receiving psychopharmacological treatment, most commonly antidepressants and benzodiazepines, with some also prescribed antipsychotics and mood stabilizers. Importantly, BPD participants were required to have a history of at least two serious suicide attempts, including at least one within the six months preceding study enrolment [19]. Both publications by Thomas et al. included in this review had largely overlapping samples (first: 44 BPD, average age: 29.5 and 44 HC, average age: 29.7, 37 females and 7 males in both; second: 41 BPD, average age: 30.4 and 41 HC, average age: 30.7, 35 females and 6 males in both), and Thomas et al.’s first publication used the same sample as Knoblich et al. [29,31,33]. Most of the BPD patients were smokers, habitual drinkers, and had been taking unspecified medications. The average age did not differ significantly between groups. Other comorbidities have not been reported in these studies. In this section, we will report only the publication concerning BDNF by Thomas et al., and the other two will be presented in scope in the next section [33].

Perroud et al. reported significantly higher methylation in both CpG regions in BPD compared with HC. Group differences remained significant after adjusting for age and sex. Moreover, methylation levels correlated with the number of childhood adversities, impulsivity, Beck Depression Inventory (BDI) scores, and hopelessness scores. After four weeks of intensive dialectical-behavioral therapy (I-DBT), methylation levels increased significantly, mainly among non-responders to treatment. Changes in methylation also correlated with changes in impulsivity, BDI, and scores of hopelessness. Interestingly, circulating BDNF protein levels were not linked to methylation in the studied regions; however, they showed an inverse relationship with the response to psychotherapy [18].

Jamshidi et al. similarly found higher mean methylation levels in BPD compared with HC in the examined region. Additionally, three CpG sites (chr11: 27744363, 27744049, 27723245) showed significantly higher methylation in BPD, whereas one CpG site (chr11: 27723218) showed significantly lower methylation. Mean methylation levels were further correlated with the severity of past suicide attempts [19].

Thomas et al. demonstrated that both mean methylation and methylation across all examined CpG sites in DNA extracted from BPD subjects were significantly higher than in HC subjects. This result remained unchanged after correction for smoking-related behaviours and early life stress [33]. Notably, neither of these covariates was significantly associated with methylation, which contrasts with the findings reported by Perroud et al. [18]. Additionally, no significant group differences or correlations were observed for DNA derived from blood samples, contrary to Perroud et al.’s findings [18,33]. This discrepancy may stem from methodological differences in methylation analysis (pyrosequencing in Thomas et al. versus high-resolution melt assay in Perroud et al.). Furthermore, methylation levels at selected CpG sites in blood-derived DNA did not correlate with those measured in saliva-derived DNA, underscoring the importance of choosing the appropriate tissue source for BDNF analyses. Moreover, methylation levels in saliva significantly decreased after 12 weeks of psychotherapy, although this effect was limited to one CpG site (chr11:2772314) and the mean methylation level. At the same time, the authors noted variability in methylation trajectory across patients: in 7 individuals, methylation remained unchanged; in 14, it decreased; and in 5, it increased. These changes in methylation did not correlate with symptom reduction following psychotherapy, again in contrast to the results reported by Perroud et al. [18,33].

### 3.7. Other Genes

Ten studies also examined other selected genes, their functions, and clinical relevance have been summarized in Supplementary Table S2. One of these, by Dammann et al., has already been described in previous sections [21]. In contrast, the study by Moser et al. used the same dataset, albeit with a slight sample size difference (45 HC and 45 BPD versus 44 HC and 45 BPD), and methodology as that of Flasbeck et al. [17,26]. However, its primary goal was to evaluate a laboratory method using NR3C1 and SERT as examples. The results for NR3C1 matched those of Flasbeck et al.

Dammann et al., Teschler et al., Edelmann et al., Gescher et al., Thomas et al., Knoblich et al., Moser et al. and Groleau et al. all used whole blood as the DNA source [20,21,22,24,26,30,31,34]. Perroud et al. have used peripheral leukocytes [34].

The studies by Dammann et al. and Teschler et al. were based on the same small sample of 24 female BPD patients (average age 33 years) and 11 female HC (average age 32 years) [21,27]. Groleau et al. examined a cohort from the same broader sample as Steiger et al., with similar sample sizes and characteristics: 19 female HC, 44 female participants with BN, and 8 females with BN + BPD [20,28]. The average age for BN + BPD was not reported; however, the average age was 22.79 years across all BN participants and 22.41 years in HC [20,28]. Perroud et al. investigated a large BPD cohort (106 women, 10 men; average age, 31.5 years) compared to an ADHD group (80 men, 33 women; average age, 37.65 years) and a BD group (65 women, 57 men; average age, 45.24 years) [34]. All groups showed significant differences in average age. Edelmann et al. included 33 women and 7 men with BPD (average age: 31.6 years), 26 women and 38 men with MDD (average age: 38.6 years), and 45 women and 20 men with SAD (average age: 25.9 years) [24]. Each clinical group had its own HC group: BPD—46 women and 7 men (average age: 28.3 years); MDD—17 women and 47 men (average age: 30.8 years); SAD—34 women and 28 men (average age: 25.9 years) [24]. Gescher et al. studied exclusively female samples: 47 BPD patients (average age: 25.21 years) and 48 HC (average age: 24.71 years) [30].

Dammann et al. found significantly higher methylation of CpG4 in MAOA in BPD compared to HC, as well as significantly higher methylation of CpG1 and CpG2, but not in CpG3 or CpG4 in COMT in BPD compared to HC. Additionally, there was a trend toward higher methylation of CpG5 in MAOB in BPD. No correction for multiple comparisons was applied [21]. Thomas et al. examined two of the same four CpG sites as Dammann et al. and observed significantly lower methylation at CpG3 (chr22:19962532) within COMT in BPD patients compared with HCs. At the same time, no group differences were found for CpG4 (chr22:19962541), nor was there any interaction between the COMT Val108/158 polymorphism variants and methylation levels at the investigated CpG sites [21,29].

Teschler et al. reported PRIMA1 hypermethylation and rDNA hypomethylation across nine CpG sites in BPD versus HC [22]. Groleau et al. observed significantly higher mean methylation across the first 10 CpGs of DRD2 exon 1 (chr11:113346140–113346389) in BN + BPD compared to HC, with a trend toward higher methylation compared to BN alone [20]. However, intergroup differences were less than 1%. Moser et al. did not find significant differences in methylation of 81 CpGs and three CpG islands within SERT between BPD and HC [26]. Edelmann et al. reported significantly higher mean methylation in exon PXDN (chr2:1632996–1633597) in BPD compared with SAD and HC, but not with MDD [24]. No association was observed between PXDN methylation and the severity of childhood adversities in BPD; however, such an association was present in MDD. The authors suggested that the null findings in BPD may be due to ceiling effects, as most BPD participants reported very high levels of childhood adversities [24]. Gescher et al. identified a differentially methylated region (DMR) within the OPRK1 promoter (chr8:53252014–53252198). Average methylation across this DMR was significantly higher in BPD versus HC and showed significant negative correlations with BPD symptom severity, impulsivity, and levels of emotional and physical neglect [30].

Perroud et al. examined eight CpGs in 5HT3AR: one within a glucocorticoid response element (CpG1_I), two in the promoter (CpG2_II, CpG3_II), three in the 5’UTR (CpG1_III, CpG2_III, CpG3_III), and two in the coding region (CpG4_III, CpG5_III). Significantly lower methylation was observed at CpG1_I and CpG5_III in BPD compared to ADHD and BD, while CpG2_II, CpG3_II, CpG1_III, CpG2_III, and CpG4_III showed higher methylation. An additive effect of the rs1062613 polymorphism and BPD was observed: CC carriers exhibited higher CpG2_III methylation than CT and TT carriers, with the effect being most pronounced in individuals with BPD. Furthermore, in BPD, CpG1_I methylation correlated negatively, and CpG2_II methylation correlated positively, with the total CTQ score. Methylation levels of CpG2_II, CpG3_II, CpG2_III, and CpG5_III correlated positively with physical abuse, while CpG1_I correlated negatively with emotional neglect. Across the entire sample, CpG2_III and CpG5_III methylation mediated associations between physical abuse severity and suicidal attempts, hospitalizations, and lifetime mood episodes [34].

Knoblich et al. examined two CpG sites within APBA3 and one CpG site within MCF2. Methylation was measured before and after a 12-week DBT program. There were no statistically significant differences in the average methylation levels of APBA3 or MCF2 between BPD and HC, either before or after therapy. These results remained the same when the analysis was limited to female participants. During therapy, some participants dropped out, and ultimately 24 patients completed the program (20 women and four men; average age: 30.8 years). The average methylation levels of both APBA3 and MCF2 did not change significantly after treatment. Notably, before treatment, the average APBA3 methylation was significantly higher in patients who later responded to therapy compared to those for whom treatment was ineffective. This difference was mainly influenced by methylation at the second CpG site examined. Similar results were observed for MCF2. When the analysis was limited to women only, the differences in average APBA3 methylation between responders and non-responders disappeared; however, the difference for the second CpG site remained. Additionally, MCF2 methylation was inversely related to the global severity index after treatment [31].

### 3.8. Epigenome-Wide Association Studies

In this review, we also included three epigenome-wide association studies (EWAS). The first, conducted by Prados et al., examined the same cohort as Perroud et al. and compared a group of 88 women and seven men diagnosed with BPD (average age 32.17 years) to 59 women and 34 men with MDD (average age 41.39 years). The MDD group was specifically selected for low levels of childhood adversity due to the common co-occurrence of MDD and BPD. In this case, the MDD group was also significantly older than the BPD group and showed similar rates of comorbidity and psychopharmacological treatment patterns as described by Perroud et al. Whole blood served as the DNA source, but correction for blood cell composition was applied. The CpG sites most strongly associated with BPD in the support vector machine (SVM) analysis were cg04907664 in EFNB1, cg16637873 in SPSB2, and cg11897887, located 1500 base pairs upstream of CST9L. In univariate analysis, the most significant findings included lower methylation in BPD compared with MDD at cg04927004 near miR124-3, cg07430978 upstream of WDR60, and cg06656994 in the 5′UTR. Many of the differentially methylated CpG sites in BPD versus MDD were located on the X chromosome. SVM analysis of the association between methylation and childhood adversity (measured by total CTQ score) revealed strong relationships for 10 CpG sites near or within OCA2, MFAP2, CST9L, KCNQ2, A2ML1, NT5DC2, and RPH3AL. However, these results were not replicated in univariate analyses, which instead identified the strongest correlations with cg26196213 in IL17RA and cg04927004 upstream of miR124-3. Functional analysis revealed that the greatest methylation differences between BPD and MDD involved genes regulating apoptosis and cell death, as well as SH3 domains, EGF-like domains, GTPase activity, and cation/ion binding. Notably, the EWAS did not replicate previously reported differences in NR3C1 methylation within the same cohort. Importantly, miR124-3 regulates the expression of NR3C1 as well as EFNB1, another gene implicated in this study [25].

The second EWAS by Arranz et al. was conducted on an independent cohort divided into a discovery and replication sample. The discovery sample included 96 women with BPD and 44 female HC, while the replication sample comprised 293 women with BPD and 114 HC. Whole blood served as the DNA source, but no correction for blood cell composition was applied. Six CpG sites were found to be significantly hypomethylated in BPD compared to controls: cg10030436 in PQBP1, cg07810091 in an intragenic region, cg22713892 in ZNF41, cg02871887 in RPL10, cg24395855 in an intragenic region, and cg20156774 in TAP2. Among BPD participants with high levels of childhood adversity, methylation levels at five CpG sites were significantly lower compared to those with low adversity and controls: cg15948871 in POUF1, cg24915915 in GPR55, cg04511534 in GGT6, cg00253346 in TNFRSF13C, and cg05478172 in FAM113B. Notably, five of these six CpG sites are located on the X chromosome. Gene set enrichment analysis showed that genes differentially methylated between BPD and controls were enriched for functions related to neurogenesis, neuronal and cellular differentiation, and cellular transport. No significant methylation differences were observed between BPD individuals with high versus low trauma exposure. In the replication cohort, only two CpG sites from the discovery phase showed significantly lower methylation in BPD compared to controls: cg10030436 in PQBP1 and cg02871887 in RPL10. Additionally, in this sample, comparing BPD patients with high childhood adversity to those with low adversity and controls revealed significantly lower methylation at cg10888111 in PXDN [23].

There was also an EWAS by Teschler et al. based on a very small sample (24 BPD, 11 HC). This study, using the 27K bead chip, identified significantly higher methylation of APBA3 (cg20366831), KCNQ1 (cg17820828), MCF2 (cg21557231), and NINJ2 (cg20781967) in BPD compared to HC, and confirmed these findings with pyrosequencing [27].

## 4. Discussion

In this systematic review, we conducted a detailed analysis of existing studies on DNA methylation changes associated with borderline personality disorder (BPD) (summarized in Table 3 for candidate genes in BPD compared to HC). The included studies were heterogeneous in terms of methodology, sample characteristics, and genes studied. Although they reported multiple differences in the epigenetic profiles of BPD compared to both psychiatric populations and healthy controls, the findings rarely formed a consistent picture.

**Table 3:** Generalized summary of findings from candidate gene methylation studies in Borderline Personality Disorder (BPD). The table summarizes generalized results of studies assessing DNA methylation differences in candidate genes among individuals with BPD compared to healthy controls (HC), and correlations within the BPD group. “BPD vs. HC” indicates direction of group differences; subsequent columns report correlations between methylation and clinical or environmental variables in BPD. ↑—higher methylation in BPD vs. HC or positive correlation; ↓—lower methylation in BPD vs. HC or negative correlation; (=)—no significant difference or association; symbols enclosed in * * indicate that the given association was investigated in more than one study; findings refer to candidate gene approaches only, excluding EWAS. Abbreviations: BPD—Borderline Personality Disorder; HC—Healthy Controls; BDNF—Brain-Derived Neurotrophic Factor; NR3C1—Glucocorticoid Receptor Gene; FKBP5—FK506 Binding Protein 5; DRD2—Dopamine D2 Receptor Gene; HTR3A—Serotonin Receptor 3A; COMT—Catechol-O-Methyltransferase; OPRK1—Opioid Receptor Kappa 1; PRIMA1—Proline-Rich Membrane Anchor 1; PXDN—Peroxidasin; rDNA—Ribosomal DNA.

Loci	BPD vs. HC	Global Severity	BPD Symptoms	Depressive Symptoms	Anxiety Symptoms	Impulsivity	Dissociative Symptoms	Emotional Abuse	Physical Abuse	Sexual Abuse	Emotional Neglect	Physical Neglect	Total Trauma Score	Number of Adversities
NR3C1 (exon 1F)	*↓/↑*		↑	(=)	(=)			*↓/(=)*	*↑/(=)*	*↑/(=)*	*↑(/=)*	*↑/(=)*	*(=)*	↑
NR3C1 (exon 1H)	↓													
NR3C1 (exon 1C)	↑													
FKBP5 (intron 7)	(=)	↑		(=)	↑			(=)	(=)	(=)	(=)	(=)	(=)	
BDNF (exon 1)	*↑*			↑		↑								
BDNF (exon 4)	*↑/(=) blood ↑ saliva*			↑		↑							(=)	
OPRK1	↓		↓			↓	(=)	(=)	(=)	(=)	↓	↓		
MAOA	↑													
MAOB	(=)													
COMT	*↓/↑*												(=)	
ABPA3	(=)	(=)												
MCF2	(=)	↓												
PXDN								(=)	(=)	(=)	(=)	(=)	(=)	(=)
5HTR2A	(=)													
5HT3AR (response element)								(=)	(=)	(=)	↓	(=)	↓	
5HT3AR (promotor)								(=)	↑	(=)	(=)	(=)	↑	
5HT3AR (body)								(=)	↑	(=)	(=)	(=)	(=)	(=)
DRD2	↑													
PRIMA1	↑													
rDNA	↓													

Most research has focused on NR3C1 and BDNF, with additional reports involving a wide range of other genes. For NR3C1, evidence indicates hypermethylation in exon 1F in BPD compared to HC, although group differences were often minimal. This hypermethylation appears to be related to certain types of childhood adversities, including physical abuse, sexual abuse, and emotional neglect; however, the pattern of associations has not been reliably replicated across studies. The only findings consistently replicated across studies without discrepancies were hypermethylation of BDNF exon 1 and the absence of a correlation between NR3C1 exon 1F methylation and total CTQ score. All other results were either investigated only once or yielded conflicting outcomes across the studies included in this review. Additionally, there is no evidence of altered methylation in FKBP1, a gene functionally related to NR3C1. Regarding BDNF, findings generally suggest hypermethylation, although results become inconsistent when different DNA sources are considered. The connection between BDNF methylation, childhood adversities, and the impact of DBT remains inconclusive. Findings for COMT are also inconsistent, with one study reporting hypermethylation and another reporting hypomethylation at different CpG sites. Other genes potentially showing hypermethylation include those involved in monoaminergic neurotransmission (MAOA, DRD, SERT, 5HT3AR), cholinergic signaling (PRIMA1), extracellular matrix (PXDN), and the endocannabinoid system (OPRK1). Hypomethylation has been observed in other CpG sites within the 5HT3AR gene. Additionally, increased methylation of APBA3 may serve as a potential biomarker for a favorable response to psychotherapy.

Further complexity arises because findings from candidate-gene studies have not been reflected in epigenome-wide association studies (EWAS), which did not find significant changes in the same genes, except for PXDN, ABPA2, and MCF2. Although EWAS identified significant methylation differences, none of the three studies produced overlapping results. Moreover, loci reaching statistical significance in EWAS were more often hypomethylated rather than hypermethylated, contrasting with candidate-gene findings. Despite numerous reports of epigenetic modifications in BPD, the current evidence base does not permit firm conclusions.

When interpreting the present findings, it is essential to acknowledge the methodological limitations of the included studies. The overall quality was moderate, but there was significant variability across different designs and analytical methods. Many reports lacked detailed explanations of participant recruitment, exclusion criteria, and dropouts, which raises concerns about selection bias. Furthermore, inconsistent control matching and incomplete adjustment for confounding factors such as medication, smoking, and trauma severity may have affected the reported methylation effects. These issues highlight the need for transparent participant tracking and the adoption of standardized methodological and reporting frameworks in future epigenetic research on BPD.

Epigenetic mechanisms provide a convincing theoretical basis for connecting environmental stressors to psychopathology, especially in BPD [44]. In general, epigenetic modifications—such as DNA methylation, histone tail modifications, and non-coding RNAs—can durably influence gene expression without altering the genetic code [32]. For example, the functional consequences of methylation at NR3C1 alternative first exons provide a mechanistic explanation for the stress axis dysregulation observed in BPD and other trauma-related disorders. Pioneering work by McGowan et al. demonstrated that individuals exposed to childhood abuse exhibited increased DNA methylation at the promoter region of exon 1F of the NR3C1 gene in hippocampal tissue, which was associated with reduced expression of GR mRNA transcripts containing exon 1F [45]. This finding suggests that hypermethylation in this region downregulates NR3C1 transcription, thereby impairing negative feedback regulation of the HPA axis. Labonté et al. extended this analysis to other promoter regions. They showed that in hippocampi of individuals with histories of childhood abuse, methylation of exon 1B and 1C promoters was negatively correlated with GR expression. In contrast, methylation of exon 1H was positively associated with its expression [46]. Taken together, these studies suggest that hypermethylation of exons 1F and 1C and hypomethylation of 1H converge functionally to reduce GR availability, potentially leading to blunted HPA reactivity and elevated baseline cortisol—a pattern often reported in BPD cohorts [10].

Interestingly, a meta-analysis of DNA methylation in the BDNF and NR3C1 genes in the context of depression showed that BDNF hypermethylation may be associated with the risk of MDD in Asian populations, whereas NR3C1 hypermethylation was linked to depressive symptoms but not to MDD itself [4]. This may suggest not only population-specific effects but also a transdiagnostic role of hypermethylation, which could be particularly relevant given the complex psychopathology of BPD. Consistently, similar links between BDNF methylation and depressive symptoms, suicidal behavior, and impulsivity were also observed in BPD populations by Perroud et al. [18]. However, the studies included in our review did not report comparable correlations with symptomatology for NR3C1 [15,16,28].

There is also a possibility that similar NR3C1 methylation patterns in BPD and MDD do not reflect a broader transdiagnostic phenomenon linked to childhood adversity but rather stem from the high comorbidity of MDD within the BPD populations studied. Perroud et al. observed nominal hypermethylation of NR3C1 exon 1F in BPD compared to MDD and MDD + PTSD groups, but they did not perform formal statistical comparisons between these diagnostic categories. Moreover, over 73% of participants in the BPD group met criteria for MDD, which complicates the interpretation of these findings [15]. A similar issue applies to the study by Flasbeck et al., which reported statistically significant hypomethylation of exon 1F in BPD relative to HC; however, just over half of the BPD participants also met diagnostic criteria for MDD [17]. Although Dammann et al. reported hypermethylation of NR3C1 1F in a sample of women diagnosed with BPD and low MDD comorbidity compared to HC, the study had a small sample size [21]. Comparable limitations apply to the study by Steiger et al., who found hypermethylation in exon 1C and hypomethylation in exon 1H in BPD relative to HC. Yet, all BPD participants also met criteria for bulimia nervosa [28]. BPD samples across the reviewed studies also exhibited substantial comorbidities with bipolar disorder, substance use disorders, and even schizoaffective disorder [15,17,21,28]. Given this diagnostic heterogeneity and methodological variation, it is difficult to determine which disorder was the true source of the observed epigenetic signal.

Variation in tissue sources may also contribute to discrepancies, as peripheral blood versus saliva can produce different methylation patterns due to tissue-specific epigenetic profiles [47]. Indeed, the study reporting lower NR3C1 methylation in BPD used saliva-derived DNA, while others using blood-based assays observed increases, indicating a possible matrix effect [15,16,17,28]. Additionally, the methods used in the laboratory (such as genome-wide arrays versus targeted bisulfite pyrosequencing) and sample size limitations influence the detection of differences [23].

Crucially, childhood trauma itself seems to be a primary factor behind epigenetic differences: its presence links to NR3C1 hypermethylation and could increase group disparities [16], whereas studies that did not focus on maltreatment (or examined loci less affected by stress) often find weaker effects. Findings on FKBP5 methylation in BPD highlight the need for careful interpretation. Flasbeck et al. found no significant differences in FKBP5 methylation between BPD patients and healthy controls. However, within BPD groups, epigenetic variation in FKBP5 has been linked to the severity of psychopathology. In the Flasbeck study, higher methylation at an intron 7 FKBP5 locus (which regulates glucocorticoid receptor sensitivity) was associated with increased anxiety symptoms and greater overall symptom severity [17]. Notably, these FKBP5 methylation correlations appeared independently of childhood maltreatment levels, suggesting that FKBP5 epigenetic dysregulation may reflect ongoing stress-related pathology in BPD rather than just a trauma footprint. Similarly, NR3C1 hypermethylation has been linked to more severe BPD symptoms [17].

Studies on *BDNF* gene methylation in individuals with BPD have shown inconsistent results, highlighting several possible reasons for these differences. Some reports indicated significantly higher methylation levels of *BDNF* promoter regions in BPD patients compared to controls, suggesting epigenetic suppression of this gene [18]. Others, however, did not confirm such differences. For example, Thomas et al. observed increased BDNF methylation only in saliva-derived DNA, not in blood, highlighting the importance of tissue selection in epigenetic research [33]. Discrepancies between studies may arise from multiple factors: analysis of different CpG sites (testing distinct BDNF promoters/exons—exon I vs. IV), varied DNA sources (peripheral blood or saliva), methodological differences (different methylation assays), and sample size and cohort composition. Additionally, studies included relatively small BPD groups that varied in symptom severity or comorbidity, making comparisons difficult.

Similarly to NR3C1, the co-occurrence of other psychiatric disorders (e.g., depression, PTSD, eating disorders) is especially important in the case of BDNF methylation changes [4,48,49]. This suggests that the epigenetic phenotype attributed to BPD may reflect a broader effect of trauma and comorbid psychopathology [48,50]. BPD patients with a history of maltreatment exhibit higher BDNF methylation, and the severity of traumatic experiences is associated with increased methylation levels of this gene [18]. This aligns with broader evidence showing that in diverse populations exposed to early stress—from women exposed to domestic violence to war veterans—increased BDNF methylation occurs in peripheral tissues [26,49]. As with NR3C1, the studies examining BDNF methylation levels in BPD included substantial comorbidity with MDD and other disorders. In the study by Perroud et al., more than 70% of participants diagnosed with BPD also met criteria for MDD, and an additional 19% had a diagnosis of BD [18]. In the case of Jamshidi et al., comorbidity with these disorders was reported in over 42% and over 8% of participants, respectively [19]. Such a high prevalence of affective disorders considerably complicates the interpretation of the findings. In contrast, Thomas et al. did not report similar comorbidities in their BPD sample, which is noteworthy given that their study failed to replicate the previously observed hypermethylation in blood-derived DNA [33].

An important question concerns how psychotherapy might reverse these epigenetic changes. Early evidence suggests that effective psychotherapy could influence BDNF methylation in BPD patients. Perroud et al. observed that, after an intensive 4-week DBT course, the average level of BDNF methylation increased in the overall patient group; however, this increase was primarily due to individuals who did not exhibit clinical improvement. By contrast, patients who responded positively to therapy exhibited a *decrease* in *BDNF* methylation—suggesting that symptom reduction was accompanied by partial reversal of stress-related epigenetic marks [18]. Similar results were reported by Thomas et al., who observed a significant decrease in BDNF promoter methylation in saliva after 12 weeks of psychotherapy in BPD patients. Overall, these findings support the idea that psychotherapy may partly “normalize” stress-related epigenetic markers in borderline disorder, although this effect appears to depend on actual clinical improvement [33]. It is important to note that these findings are preliminary and subject to several limitations. First, there is a lack of replication in larger, independent groups—existing studies vary in protocols (tissue, CpG sites, laboratory methods) and patient profiles, which makes it hard to draw definitive conclusions. Second, BDNF methylation changes are not specific to BPD— as noted, they also happen in other disorders and stress-related states— therefore, increased BDNF methylation might indicate a general response to chronic stress or mood issues rather than a specific sign of BPD. Finally, no clear link has yet been established between *BDNF* methylation levels and peripheral BDNF protein expression [18], leaving open the question of the functional meaning of these findings. All these factors may contribute to inconsistent study results and highlight the need for further systematic research—especially longitudinal, large-scale studies that consistently control for trauma, smoking, and medication, while applying standardized measurement methods. Such approaches will help clarify whether *BDNF* methylation serves as a marker of vulnerability to BPD and its symptoms, or rather reflects environmental factors, and whether it can be used as a biological indicator of treatment response in this disorder.

Numerous other candidate gene studies suggest that BPD is linked to abnormal DNA methylation patterns, often characterized by hypermethylation at specific gene promoters in blood; however, most of these findings are based on single studies. For example, BPD patients have shown higher methylation in several genes involved in monoamine neurotransmission, including the serotonin receptor genes *HTR2A* and *HTR3A*, the dopamine D_2_ receptor gene *DRD2*, and enzymes like *MAOA*, *MAOB*, and *COMT* that break down monoamine neurotransmitters [20,26,27,29,34]. Consistent with this, women with a bulimia-spectrum disorder who also had BPD showed significantly higher *DRD2* promoter methylation than those without BPD, and a trend toward *DRD2* hypermethylation was also observed among individuals reporting childhood sexual abuse [20]. Early-life trauma seems to play a significant role in these epigenetic differences: in one study, exposure to childhood maltreatment (especially physical abuse) was linked to widespread increased methylation across *HTR3A* CpG sites, and this hypermethylation mediated higher clinical severity, such as more suicide attempts, hospitalizations, and recurrence of mood episodes [34]. Similarly, *PRIMA1* (a gene encoding a protein that anchors acetylcholinesterase at neuronal membranes) was found to be about 1.6 times more methylated in BPD compared to controls [22]. Since *PRIMA1* methylation can reduce acetylcholinesterase activity and increase cholinergic signaling, this finding supports reports of overactive cholinergic systems in BPD and suggests a mechanistic link between epigenetic modifications and the cognitive-affective symptoms of the disorder [22]. Conversely, some studies have identified hypomethylation in BPD at certain sites; for instance, Teschler et al. reported significantly lower methylation (~10% decrease) of ribosomal DNA (*rDNA*) repeats in the blood of BPD patients, even though increased *rDNA* methylation was observed in the brain tissue of suicide victims [22].

EWAS findings in BPD have identified a few notable loci, but results vary across studies. For example, Teschler et al. reported small but significant hypermethylation (~5–10% higher) at CpG sites in APBA2 and MCF2 in the blood of BPD patients compared to controls [27]. These differences were modest in size and came from a small sample. Subsequent studies have struggled to replicate these specific gene hits. For instance, Knoblich et al. re-examined APBA3 (a gene related to APBA2) and MCF2 in a larger cohort and found no significant case–control methylation differences at these loci [31]. Interestingly, that study instead found that higher baseline methylation in APBA3 and MCF2 predicted better psychotherapy response—BPD patients who eventually responded to dialectical behaviour therapy showed significantly higher pre-treatment methylation at these genes than non-responders. This suggests that while APBA2/3 and MCF2 may not be reliable diagnostic markers of BPD across populations, their methylation could be linked to clinical features such as treatment outcome [31].

Recent EWAS have highlighted PXDN (peroxidasin) in relation to early-life trauma. Arranz et al. identified a CpG site in PXDN (cg10888111) that was hypomethylated in BPD patients with a history of childhood maltreatment compared to both BPD patients without trauma and healthy controls [23]. This PXDN locus also exhibited a significant methylation difference between BPD patients and controls in a large replication sample, highlighting its potential importance [23]. These findings were only partly replicated by Edelmann et al., who found significantly higher methylation in BPD compared to HC and SAD, but not MDD. Additionally, methylation levels correlated with childhood adversities only in the MDD group, not in BPD [24]. Notably, in that study, high-adversity groups showed hypermethylation (not hypo-) at PXDN compared to low-adversity groups. The direction of the association was thus reversed compared to Arranz et al.’s BPD-only sample, which the authors suggest could be due to differences in cohorts or disorder context. This discrepancy illustrates how EWAS findings can differ and underscores the need for further replication [23,24]. Overall, the mixed results for genes like PXDN, APBA2, and MCF2 probably reflect methodological and sample differences. Early BPD EWAS had limited power and used the 27K methylation array, which covers relatively few CpGs (mostly in promoters). Newer studies with 450K/850K arrays offer wider coverage, but even then, effect sizes of methylation differences in BPD tend to be small. Tissue source is another factor: all cited EWAS examined peripheral blood DNA, which may not show brain-specific methylation changes important for BPD pathology. Variation in clinical features (e.g., different levels of childhood trauma or symptom severity among samples) can further influence results, as seen with PXDN, where an adversity effect appeared in one BPD group and in a transdiagnostic sample but was absent or opposite in other comparisons [23,24]. The lack of consistent replication for many hits (including the *APBA2*/*APBA3* and *MCF2* findings) underscores caution. It suggests that some initial EWAS signals may have been false positives or specific to the original sample’s context. In summary, while epigenome-wide screens have highlighted *PXDN*, *APBA2*/*APBA3*, and *MCF2* as tentative epigenetic markers in BPD, linking DNA methylation to early trauma exposure or clinical outcomes, these findings remain provisional. Future research with larger cohorts, careful replication, and possibly tissue-specific approaches will be crucial for determining which methylation changes are truly robust in BPD and for clarifying the biological or environmental factors driving the inconsistencies in current EWAS results.

The clinical utility of DNA methylation markers in BPD remains uncertain at this stage, particularly with regard to their use in risk prediction or trait-level diagnosis. The difficulty in distinguishing between transdiagnostic effects of trauma and BPD-specific methylation signatures, combined with high rates of affective comorbidities in most included samples, limits the specificity of the observed findings. Moreover, in the case of the most extensively studied loci—such as BDNF and NR3C1—the magnitude of methylation differences between BPD and healthy controls appears modest, potentially precluding adequate sensitivity or specificity for diagnostic applications. Nevertheless, preliminary evidence suggests that methylation levels at certain loci (e.g., BDNF, APBA3, MCF2) may hold promise as state markers for psychotherapy response or treatment sensitivity, particularly in the context of dialectical behavior therapy (DBT). These findings require further validation, however, and a critical next step is to establish the baseline directionality and magnitude of methylation changes in BPD before considering their implementation in treatment monitoring frameworks.

This systematic review has several limitations. First, although a comprehensive search strategy was employed, the review was limited to studies published in peer-reviewed journals and available in English, which may have introduced publication and language biases. Second, the included studies were highly variable regarding sample characteristics (such as sex distribution, psychiatric comorbidities, and trauma exposure), DNA sources (e.g., blood, saliva), and laboratory methods (e.g., pyrosequencing, bisulfite sequencing, conversion efficiency), making direct comparisons difficult. Third, most studies relied on small sample sizes, which limited statistical power and increased the risk of both type I and type II errors. Fourth, only a few studies controlled for potential confounders such as medication use, smoking status, or cellular heterogeneity in DNA sources. Fifth, even among studies focused on the same candidate gene, different promoter regions or CpG sites were often examined, and many failed to report effect sizes or unadjusted methylation levels, restricting the ability to extract comparable data. Finally—and most notably—due to the limited number of studies investigating the same gene regions with similar methodologies, conducting a quantitative synthesis like a meta-analysis was not possible. Therefore, evaluating heterogeneity, risk of bias, or sensitivity was not feasible, and our findings should be understood within the context of a qualitative systematic review.

## 5. Conclusions

This systematic review summarizes current evidence on DNA methylation alterations in BPD, including frequently studied loci such as NR3C1 and BDNF. Nonetheless, substantial methodological heterogeneity, small sample sizes, and inconsistent replication substantially constrain the interpretability and translational value of existing findings. Progress in the field will require larger, longitudinal studies with more precisely defined clinical phenotypes and reduced psychiatric comorbidity, particularly with adequate adjustment for affective symptom severity. More systematic inclusion of male participants is also needed. Clarifying the functional consequences of reported methylation differences—especially their effects on gene expression and protein levels—remains essential. Future research should additionally investigate treatment-related changes in methylation, compare peripheral tissues (e.g., saliva versus blood), and improve CpG site coverage within candidate regions. Ultimately, integrating genetic, epigenetic, and environmental measures within multi-omics designs will be necessary to evaluate whether methylation markers have any potential as predictors of clinical course, treatment response, or neurobiological vulnerability in BPD.

## Supplementary Materials

The following supporting information can be downloaded at: https://www.mdpi.com/article/10.3390/jcm14228182/s1. Table S1: Psychometric instruments used in included studies. Table S2: Other genes included in this review. PRISMA 2020 Main Checklist. PRIMSA Abstract Checklist. Informations based on NHS Genes. Refs. [51,52,53,54,55,56,57,58,59,60,61,62,63,64] are cited in the supplementary.

## Abbreviations

The following abbreviations are used in this manuscript:

BPD	Borderline Personality Disorder
HPA	Hypothalamic–pituitary–adrenal axis
BDNF	Brain-derived neurotrophic factor
MeSH	Medical Subject Headings
EWAS	Epigenome-wide association studies
HC	Healthy controls
MDD	Major Depressive Disorder
SAD	Social Anxiety Disorder
DE	Depressive Episode
ME	Mood Episode
PME	Psychotic Mood Episode
CTQ	Childhood Trauma Questionnaire
CTI	Childhood Trauma Adversities
FT	Fagerstrom Test
COBRA	Combined Bisulfite Restriction Analysis
BN	Bulimia Nervosa
ED	Eating Disorder
PTSD	Posttraumatic Stress Disorder
GR	Glucocorticoid Receptor
DBT	Dialectical-Behavioral Therapy
