# Supplementary material for: The Epigenetic Landscape of Borderline Personality Disorder: Insights from a Systematic Review
Source: J Clin Med. 2025 Nov 18;14(22):8182. doi: 10.3390/jcm14228182 (PMC12653379; doi:10.3390/jcm14228182)
Supplement: Supplementary file 1 [file jcm-14-08182-s001.zip › jcm-3876957-supplementary.pdf]

PRISMA 2020 Main Checklist

Topic	No.	Item	Location where item is reported
**TITLE**			
**Title**	1	Identify the report as a systematic review.	In the title. abstract (methods), introduction (lat paragraph)
**ABSTRACT**			
**Abstract**	2	See the PRISMA 2020 for Abstracts checklist	
**INTRODUCTION**			
**Rationale**	3	Describe the rationale for the review in the context of existing knowledge.	Introduction
**Objectives**	4	Provide an explicit statement of the objective(s) or question(s) the review addresses.	Introduction
**METHODS**			
**Eligibility criteria**	5	Specify the inclusion and exclusion criteria for the review and how studies were grouped for the syntheses.	Materials and methods (3d paragraph)
**Information sources**	6	Specify all databases, registers, websites, organisations, reference lists and other sources searched or consulted to identify studies. Specify the date when each source was last searched or consulted.	Materials and methods (2nd paragraph)
**Search strategy**	7	Present the full search strategies for all databases, registers and websites, including any filters and limits used.	Materials and methods (2nd paragraph)
**Selection process**	8	Specify the methods used to decide whether a study met the inclusion criteria of the review, including how many reviewers screened each record and each report retrieved, whether they worked independently, and if applicable, details of automation tools used in the process.	Materials and methods (Data Extraction)
**Data collection process**	9	Specify the methods used to collect data from reports, including how many reviewers collected data from each report, whether they worked independently, any processes for obtaining or confirming data from study investigators, and if applicable, details of automation tools used in the process.	Materials and methods (Data Extraction)

Topic	No.	Item	Location where item is reported
**Data items**	10a	List and define all outcomes for which data were sought. Specify whether all results that were compatible with each outcome domain in each study were sought (e.g. for all measures, time points, analyses), and if not, the methods used to decide which results to collect.	Materials and methods (Data Extraction)
	10b	List and define all other variables for which data were sought (e.g. participant and intervention characteristics, funding sources). Describe any assumptions made about any missing or unclear information.	Materials and methods (Data Extraction)
**Study risk of bias assessment**	11	Specify the methods used to assess risk of bias in the included studies, including details of the tool(s) used, how many reviewers assessed each study and whether they worked independently, and if applicable, details of automation tools used in the process.	Materials and methods (Quality assessment)
**Effect measures**	12	Specify for each outcome the effect measure(s) (e.g. risk ratio, mean difference) used in the synthesis or presentation of results.	Materials and methods (Data Extraction)
**Synthesis methods**	13a	Describe the processes used to decide which studies were eligible for each synthesis (e.g. tabulating the study intervention characteristics and comparing against the planned groups for each synthesis (item 5)).	Materials and methods (Data Extraction)
	13b	Describe any methods required to prepare the data for presentation or synthesis, such as handling of missing summary statistics, or data conversions.	Not applicable
	13c	Describe any methods used to tabulate or visually display results of individual studies and syntheses.	Not applicable
	13d	Describe any methods used to synthesize results and provide a rationale for the choice(s). If meta-analysis was performed, describe the model(s), method(s) to identify the presence and extent of statistical heterogeneity, and software package(s) used.	Materials and methods (Data Extraction)
	13e	Describe any methods used to explore possible causes of heterogeneity among study results (e.g. subgroup analysis, meta-regression).	Not applicable
	13f	Describe any sensitivity analyses conducted to assess robustness of the synthesized results.	Not applicable
**Reporting bias assessment**	14	Describe any methods used to assess risk of bias due to missing results in a synthesis (arising from reporting biases).	Materials and methods (Quality assessment)
**Certainty assessment**	15	Describe any methods used to assess certainty (or confidence) in the body of evidence for an outcome.	No applicable

Topic	No.	Item	Location where item is reported
**RESULTS**			
**Study selection**	16a	Describe the results of the search and selection process, from the number of records identified in the search to the number of studies included in the review, ideally using a flow diagram.	Results (Study selection, Figure 1)
	16b	Cite studies that might appear to meet the inclusion criteria, but which were excluded, and explain why they were excluded.	Not applicable
**Study characteristics**	17	Cite each included study and present its characteristics.	Table 1
**Risk of bias in studies**	18	Present assessments of risk of bias for each included study.	Results (Quality assesment)
**Results of individual studies**	19	For all outcomes, present, for each study: (a) summary statistics for each group (where appropriate) and (b) an effect estimate and its precision (e.g. confidence/credible interval), ideally using structured tables or plots.	Results
**Results of syntheses**	20a	For each synthesis, briefly summarise the characteristics and risk of bias among contributing studies.	Results (Quality assesment)
	20b	Present results of all statistical syntheses conducted. If meta-analysis was done, present for each the summary estimate and its precision (e.g. confidence/credible interval) and measures of statistical heterogeneity. If comparing groups, describe the direction of the effect.	Not applicable
	20c	Present results of all investigations of possible causes of heterogeneity among study results.	Results, discussion
	20d	Present results of all sensitivity analyses conducted to assess the robustness of the synthesized results.	Not applicable
**Reporting biases**	21	Present assessments of risk of bias due to missing results (arising from reporting biases) for each synthesis assessed.	Not applicable
**Certainty of evidence**	22	Present assessments of certainty (or confidence) in the body of evidence for each outcome assessed.	Not applicable
**DISCUSSION**			
**Discussion**	23a	Provide a general interpretation of the results in the context of other evidence.	Discussion (1st and 2nd paragraphs)
	23b	Discuss any limitations of the evidence included in the review.	Discussion

Topic	No.	Item	Location where item is reported
	23c	Discuss any limitations of the review processes used.	Discussion (Last paragraph)
	23d	Discuss implications of the results for practice, policy, and future research.	Conclusions
**OTHER INFORMATION**			
**Registration and protocol**	24a	Provide registration information for the review, including register name and registration number, or state that the review was not registered.	Materials and Methods (1st paragraph)
	24b	Indicate where the review protocol can be accessed, or state that a protocol was not prepared.	Materials and Methods (1st paragraph)
	24c	Describe and explain any amendments to information provided at registration or in the protocol.	Not applicable
**Support**	25	Describe sources of financial or non-financial support for the review, and the role of the funders or sponsors in the review.	Funding
**Competing interests**	26	Declare any competing interests of review authors.	Conficts of Intrest
**Availability of data, code and other materials**	27	Report which of the following are publicly available and where they can be found: template data collection forms; data extracted from included studies; data used for all analyses; analytic code; any other materials used in the review.	Not applicable

PRIMSA Abstract Checklist

Topic	No.	Item	Reported?
**TITLE**			
**Title**	1	Identify the report as a systematic review.	Yes
**BACKGROUND**			
**Objectives**	2	Provide an explicit statement of the main objective(s) or question(s) the review addresses.	Yes
**METHODS**			
**Eligibility criteria**	3	Specify the inclusion and exclusion criteria for the review.	Yes

Topic	No.	Item	Reported?
**Information sources**	4	Specify the information sources (e.g. databases, registers) used to identify studies and the date when each was last searched.	Yes
**Risk of bias**	5	Specify the methods used to assess risk of bias in the included studies.	Yes
**Synthesis of results**	6	Specify the methods used to present and synthesize results.	Yes
**RESULTS**			
**Included studies**	7	Give the total number of included studies and participants and summarise relevant characteristics of studies.	Yes
**Synthesis of results**	8	Present results for main outcomes, preferably indicating the number of included studies and participants for each. If meta-analysis was done, report the summary estimate and confidence/credible interval. If comparing groups, indicate the direction of the effect (i.e. which group is favoured).	Yes
**DISCUSSION**			
**Limitations of evidence**	9	Provide a brief summary of the limitations of the evidence included in the review (e.g. study risk of bias, inconsistency and imprecision).	Yes
**Interpretation**	10	Provide a general interpretation of the results and important implications.	Yes
**OTHER**			
**Funding**	11	Specify the primary source of funding for the review.	Yes
**Registration**	12	Provide the register name and registration number.	Yes

From: Page MJ, McKenzie JE, Bossuyt PM, Boutron I, Hoffmann TC, Mulrow CD, et al. The PRISMA 2020 statement: an updated guideline for reporting systematic reviews. MetaArXiv. 2020, September 14. DOI: 10.31222/osf.io/v7gm2. For more information, visit: www.prisma-statement.org

Supplementary Table 1: Psychometric instruments used in included studies.

Psychometric tool	Description
International Personality Disorder Examination (IPDE) [1]	A semi-structured clinical interview developed by the WHO for the reliable assessment and diagnosis of personality disorders according to ICD-10 and DSM-IV criteria. It provides both categorical diagnoses and dimensional ratings, allowing clinicians and researchers to evaluate personality pathology with standardized methodology. Symptoms must be present for at least five years. It is not appropriate for clients with severe depression, psychosis, low intelligence or cognitive impairment.
Revised Diagnostic Interview for Borderlines (DIB-R) [2]	A semi-structured interview designed specifically to assess the presence and severity of borderline personality disorder. It evaluates symptoms across affective, cognitive, impulsive, and interpersonal domains, providing both categorical diagnosis and dimensional scoring.

McLean Screening Instrument for Borderline Personality Disorder (MSI-BPD) [3]	A brief, 10-item self-report questionnaire designed to screen for borderline personality disorder symptoms. It is easy to administer in clinical and research settings and demonstrates good sensitivity and specificity. While not a diagnostic tool, it is useful for identifying individuals who may require further comprehensive assessment.
Borderline Symptom List (BSL-23)[4]	A short self-report questionnaire assessing the severity of borderline personality disorder symptoms. Derived from the longer BSL-95, it covers key domains such as emotional dysregulation, self-image, and interpersonal difficulties. Widely used for monitoring symptom severity and treatment progress in both clinical and research contexts.
Beck Depression Inventory II (BDI-II)[5]	A 21-item self-report questionnaire widely used to assess the presence and severity of depressive symptoms. It evaluates emotional, cognitive, and physical aspects of depression over the past two weeks.
Beck Hopelessness Scale (BHS)[6]	A 20-item self-report questionnaire that measures negative expectations about the future. It assesses three dimensions of hopelessness: feelings about the future, loss of motivation, and future expectations. The BHS is frequently used in clinical and research settings as an indicator of suicide risk and treatment outcomes.
Barratt Impulsiveness Scale (BIS-10)[7]	A self-report questionnaire designed to measure impulsivity as a multidimensional personality trait. It assesses cognitive, motor, and non-planning aspects of impulsive behavior.
Symptom Checklist-90-Revised (SCL-90-R)[8]	A 90-item self-report questionnaire that assesses a broad range of psychological symptoms and distress. It measures nine primary dimensions, including depression, anxiety, somatization, and interpersonal sensitivity, along with global indices of distress.
Saarbrücker Persönlichkeitsfragebogen (SPF IRI)	The German adaptation of Davis's *Interpersonal Reactivity Index (IRI)*. It is a self-report questionnaire measuring empathy across four dimensions: perspective taking, fantasy, empathic concern, and personal distress.
Freeman scale (suicidal intent)[9]	A clinician-rated instrument designed to assess the severity of suicidal intent following a self-harm episode. It evaluates both the circumstances of the attempt and the individual's subjective intent, providing a structured measure of suicide risk.
Wender Utah Rating Scale (WURS)[10]	A retrospective self-report questionnaire developed to assess childhood symptoms of attention-deficit/hyperactivity disorder (ADHD) in adults. It evaluates behavioral and emotional characteristics associated with ADHD during childhood, aiding in the retrospective diagnosis of adult ADHD.
Zanarini Rating Scale for Borderline Personality Disorder (ZAN-BPD)[11]	A clinician-administered interview assessing the severity of borderline personality disorder symptoms over the past week. It covers affective, cognitive, impulsive, and interpersonal domains, providing both total and subscale scores.
Dissociative Experiences Scale (DES)[11]	A 28-item self-report questionnaire designed to assess the frequency of dissociative experiences in everyday life. It captures phenomena such as depersonalization, derealization, amnesia, and absorption, providing both a mean score and clinical cut-offs.
State-Trait Anger Expression Inventory (STAXI)[12]	A self-report questionnaire designed to assess the intensity of anger as an emotional state, the disposition to experience anger as a personality trait, and the ways anger is expressed or controlled. It provides subscales for state anger, trait anger, and anger expression/control, making it a widely used tool in both clinical and research settings.
Comprehensive Psychopathological Rating Scale [13]	A clinician-rated instrument designed to assess a wide range of psychiatric symptoms and syndromes. It provides standardized ratings across domains such as mood, anxiety, psychotic symptoms, and cognitive disturbances, allowing both dimensional and categorical evaluations.

Supplementary Table 2: Other genes included in this review. Informations based on NHS Genes.

Gene code	Gene name	Description	Role in pathophysiology

5HT3AR	5-Hydroxytryptamine (Serotonin) Receptor 3A	Serotonin receptor, enables identical protein binding activity and ligand-gated monoatomic ion channel activity involved in regulation of presynaptic membrane potential. Involved in inorganic cation transmembrane transport and serotonin receptor signaling pathway. Part of serotonin-activated cation-selective channel complex.	Alcohol dependence, Cocaine dependence, Heroin dependence, Irritable Bowel Syndrome, Nicotine Dependence
A2ML1	Alpha-2-Macroglobulin Like 1	A member of the alpha-macroglobulin superfamily, N-glycosylated monomeric protein that acts as an inhibitor of several proteases, form covalent interactions with proteases.	Paraneoplastic Pemphigus, Noonan syndrome, Otitis Media
APBA2	Amyloid Beta Precursor Protein Binding Family A Member 2	A member of the X11 protein family, neuronal adapter protein that interacts with the Alzheimer's disease amyloid precursor protein, it stabilizes APP and inhibits production of proteolytic APP fragments including the A beta peptide, involved in signal transduction processes, a putative vesicular trafficking protein in the brain that can form a complex with the potential to couple synaptic vesicle exocytosis to neuronal cell adhesion	Alzheimer's disease
APBA3	Amyloid Beta Precursor Protein Binding Family A Member 3	A member of the X11 protein family, an adapter protein that interacts with the Alzheimer's disease amyloid precursor protein, involved in signal transduction processes.	Alzheimer's disease
COMT	Catechol-O-methyltransferase	Catalyzes the transfer of a methyl group from S-adenosylmethionine to catecholamines, including dopamine, epinephrine, and norepinephrine, results in degradative pathway of the catecholamine transmitters	Metabolism of catechol drugs used in the treatment of hypertension, asthma, and Parkinson's disease
CST9L	Cystatin-9-like	A class of cysteine proteinase inhibitors	Tissue reorganization during early testis development
DRD2	Dopamine Receptor D2	D2 subtype of the dopamine receptor, G-protein coupled receptor inhibits adenylyl cyclase activity	Myoclonus Dystonia, Schizophrenia
EFNB1	Ephrin B1	A type I membrane protein, a ligand of Eph-related receptor tyrosine kinases	Cell adhesion, function in the development or maintenance of the nervous system
FAM113B/PCED1B	PC-Esterase Domain Containing 1B	A protein that belongs to the GDSL/SGNH-like acyl-esterase family - hydrolases modifying biopolymers on the cell surface	Multiple noeplasms

GGT6	Gamma-Glutamyltransferase 6	The gamma-glutamyltransferase gene family, a membrane-bound extracellular enzyme that cleaves gamma-glutamyl peptide bonds in glutathione and other peptides and transfers the gamma-glutamyl moiety to acceptors, it provides substrates for glutathione synthesis	Multiple noeplasms
GPR55	G Protein-Coupled Receptor 55	Belongs to the G-protein-coupled receptor superfamily, the encoded integral membrane protein is a cannabinoid receptor	Major Depression
IL17RA	Interleukin 17 Receptor A	Interleukin 17A receptor is a ubiquitous type I membrane glycoprotein that binds with low affinity to interleukin 17A. Interleukin 17A is a proinflammatory cytokine secreted by activated T-lymphocytes, is a inducer of the maturation of CD34-positive hematopoietic precursors into neutrophils.	Inflammatory and autoimmune diseases such as Rheumatoid Arthritis
KCNQ1	Potassium Voltage-Gated Channel Subfamily Q Member 1	A voltage-gated potassium channel required for repolarization phase of the cardiac action potential	Beckwith-Wiedemann syndrome, hereditary long QT syndrome 1, Jervell and Lange-Nielsen syndrome, Familial Atrial Fibrillation
KCNQ2	Potassium Voltage-Gated Channel Subfamily Q Member 2	The M channel is a potassium channel that plays a critical role in the regulation of neuronal excitability. The M channel is formed by the association of the protein encoded by this gene and a related protein encoded by the KCNQ3 gene, both integral membrane proteins.	Benign Familial Neonatal Convulsions type 1
MAOA	Monoamine Oxidase A	Mitochondrial enzymes which catalyze the oxidative deamination of amines	Brunner syndrome, a variety of other psychiatric disorders, including Antisocial Behavior
MAOB	Monoamine Oxidase B	The protein belongs to the flavin monoamine oxidase family, it catalyzes the oxidative deamination of biogenic and xenobiotic amines and plays an important role in the metabolism of neuroactive and vasoactive amines in the central nervous sysytem and peripheral tissues.	Parkinson's Disease, Alzheimer's disease, Prostate Cancer
MCF2	MCF.2 Cell Line Derived Transforming Sequence	The oncogenic protein, a guanine nucleotide exchange factor (GEF) that exerts control over some members of the Rho family of small GTPases.	Borderline Personality Disorder

MFAP2	Microfibril Associated Protein 2	Microfibrillar-associated protein 2 is a major antigen of elastin-associated microfibrils	Inherited Connective Tissue Diseases
miR124-3	MicroRNA 124-3	MicroRNAs are short non-coding RNAs that are involved in post-transcriptional regulation of gene expression in multicellular organisms by affecting both the stability and translation of mRNAs	Gastric Cancers, Borderline Personality Disorder, Major Depressive
NINJ2	Ninjurin 2	Belongs to the ninjurin family - for nerve injury induced. It is a cell surface adhesion protein that is upregulated in Schwann cells surrounding the distal segment of injured nerve, and promotes neurite outgrowth.	Nerve regeneration after nerve injury
NT5DC2	5'-Nucleotidase Domain Containing 2	Enable 5'-nucleotidase activitym, is involved in negative regulation of dopamine biosynthetic process, negative regulation of oxidoreductase activity and negative regulation of peptidyl-serine phosphorylation.	Multiple noeplasms
OCA2	OCA2 Melanosomal Transmembrane Protein	Human homolog of the mouse p (pink-eyed dilution) gene, an integral membrane protein involved in small molecule transport, specifically tyrosine, which is a precursor to melanin synthesis, mammalian pigmentation	Type 2 Oculocutaneous Albinism
OPRK1	Opioid Receptor Kappa 1	An opioid receptor for endogenous ligands, a receptor for synthetic opioids, plays a role in the perception of pain and mediating the hypolocomotor, analgesic and aversive actions of synthetic opioids	Alcohol Dependence, Opiate Addiction
POUF1	POU Class 1 Homeobox 1	A member of the POU family of transcription factors that regulate mammalian development, regulates expression of genes involved in pituitary development and hormone expression.	Combined Pituitary Hormone Deficiency
PQBP1	Polyglutamine Binding Protein 1	A nuclear polyglutamine-binding protein that is involved with transcription activation.	Renpenning syndrome 1 and other syndromes with X-linked cognitive disability
PRIMA1	Proline Rich Membrane Anchor 1	The product of PRIMA1 functions organize acetylcholinesterase (AChE) into tetramers, and to anchor AChE at neural cell membranes	Multiple noeplasms, Nocturnal Frontal Lobe Epilepsy, Borderline personality disorder
PXDN	Peroxidasin	A heme-containing peroxidase involved in extracellular matrix formation	Corneal Opacification and other ocular anomalies, Microphthalmia, Anterior

			Segment Dysgenesis, Fibrotic Kindey
RPH3AL	Rabphilin 3A Like (Without C2 Domains	Plays a direct regulatory role in calcium-ion-dependent exocytosis and plays a key role in insulin secretion by pancreatic cells, tumor suppressor	Multiple noeplasms, Borderline personality disorder
RPL10	Ribosomal Protein L10	A ribosomal protein that is a component of the 60S ribosome subunit	Autism Spectrum Disorders
SERT/SLC6A4	Serotonin Transporters/Solute Carrier Family 6 Member 4	An integral membrane protein that transports the neurotransmitter serotonin from synaptic spaces into presynaptic neurons, terminates the action of serotonin and recycles it in a sodium-dependent manner	Major Depression
TAP2	Transporter 2, ATP Binding Cassette Subfamily B Member	The membrane-associated protein is a member of the superfamily of ATP-binding cassette (ABC) transporters. ABC proteins transport various molecules across extra- and intra-cellular membranes. This protein is a member of the MDR/TAP subfamily, who are involved in multidrug resistance.	Ankylosing Spondylitis, Insulin-dependent Diabetes Mellitus, Celiac Disease
TNFRSF13C	TNF Receptor Superfamily Member 13C	A receptor for BAFF and is a type III transmembrane protein which is the principal receptor required for BAFF-mediated mature B-cell survival.	B-cell hyperplasia, Systemic Lupus Erythematosus
WDR60/DYNC2I1	WD Repeat Domain 60/Dynein 2 Intermediate Chain 1	A member of the WD repeat protein family, who are involved in a variety of cellular processes including cell cycle progression, signal transduction, apoptosis, and gene regulation.	The formation of cilia, Short-rib Polydactyly, Jeune syndrome
ZNF41	Zinc Finger Protein 41	A protein that contains KRAB-A and KRAB-B domains multiple zinc finger DNA binding motifs and finger linking regions characteristic of the Kruppel family.	X-linked cognitive disability

1. Magallón-Neri, E.M.; Forns, M.; Canalda, G.; Fuente, J.E.D.L.; García, R.; González, E.; Lara, A.; Castro-Fornieles, J. Usefulness of the International Personality Disorder Examination Screening Questionnaire for Borderline and Impulsive Personality Pathology in Adolescents. *Compr. Psychiatry* **2013**, *54*, 301–308, doi:10.1016/j.comppsych.2012.07.064.

2. Wall, K.; Sharp, C.; Ahmed, Y.; Goodman, M.; Zanarini, M.C. Parent–Adolescent Concordance on the Revised Diagnostic Interview for Borderlines (DIB-R) and the Childhood Interview for Borderline Personality Disorder (CI-BPD). *Pers. Ment. Heal.* **2017**, *11*, 179–188, doi:10.1002/pmh.1376.

3. Semaan, F.; Croarkin, P.E. The McLean Screening Instrument for Borderline Personality Disorder: A Review. *J. Psychiatr. Pr.* **2025**, *31*, 20–23, doi:10.1097/pra.0000000000000827.

4. Kleindienst, N.; Jungkunz, M.; Bohus, M. A Proposed Severity Classification of Borderline Symptoms Using the Borderline Symptom List (BSL-23). *Borderline Pers. Disord. Emot. Dysregulation* **2020**, *7*, 11, doi:10.1186/s40479-020-00126-6.

5. Wang, Y.-P.; Gorenstein, C. Psychometric Properties of the Beck Depression Inventory-II: A Comprehensive Review. *Rev. Bras. Psiquiatr.* **2013**, *35*, 416–431, doi:10.1590/1516-4446-2012-1048.

6. Kocalevent, R.-D.; Finck, C.; Pérez-Trujillo, M.; Sautier, L.; Zill, J.; Hinz, A. Standardization of the Beck Hopelessness Scale in the General Population. *J. Ment. Heal.* **2017**, *26*, 516–522, doi:10.1080/09638237.2016.1244717.

7. Barratt, E.S. Factor Analysis of Some Psychometric Measures of Impulsiveness and Anxiety. *Psychol. Rep.* **1965**, *16*, 547–554, doi:10.2466/pr0.1965.16.2.547.

8. Kostaras, P.; Martinaki, S.; Asimopoulos, C.; Maltezou, M.; Papageorgiou, C. The Use of the Symptom Checklist 90-R in Exploring the Factor Structure of Mental Disorders and the Neglected Fact of Comorbidity. *Psychiatry Res.* **2020**, *294*, 113522, doi:10.1016/j.psychres.2020.113522.

9. Ross, M. THE PREDICTION OF SUICIDE Edited by Aaron T. Beck, M.D., Harvey L. P. Resnik, M.D., and Dan J. Lettieri, Ph.d.; Charles Press, Bowie, Maryland, 1974, 249 Pages, $12.95. *Psychiatr. Serv.* **1975**, *26*, 45–46, doi:10.1176/ps.26.1.45.

10. Ward, M.F.; Wender, P.H.; Reimherr, F.W. The Wender Utah Rating Scale: An Aid in the Retrospective Diagnosis of Childhood Attention Deficit Hyperactivity Disorder [Published Erratum Appears in Am J Psychiatry 1993 Aug;150(8):1280]. *Am. J. Psychiatry* **1993**, *150*, 885–890, doi:10.1176/ajp.150.6.885.

11. Zanarini, M.C.; Vujanovic, A.A.; Parachini, E.A.; Boulanger, J.L.; Frankenburg, F.R.; Hennen, J. Zanarini Rating Scale For Borderline Personality Disorder (ZAN-BPD): A Continuous Measure of DSM-IV Borderline Psychopathology. *J. Pers. Disord.* **2003**, *17*, 233–242, doi:10.1521/pedi.17.3.233.22147.

12. Forgays, D.G.; Forgays, D.K.; Spielberger, C.D. Factor Structure of the State-Trait Anger Expression Inventory. *J. Pers. Assess.* **1997**, *69*, 497–507, doi:10.1207/s15327752jpa6903_5.

13. Åsberg, M.; Montgomery, S.A.; Perris, C.; Schalling, D.; Sedvall, G. A COMPREHENSIVE PSYCHOPATHOLOGICAL RATING SCALE. *Acta Psychiatr. Scand.* **1978**, *57*, 5–27, doi:10.1111/j.1600-0447.1978.tb02357.x.
